# Development of programme theory for integration of service user and caregiver involvement in mental health system strengthening: protocol for realist systematic review

**DOI:** 10.1186/s13033-018-0220-4

**Published:** 2018-07-24

**Authors:** Sisay Abayneh, Heidi Lempp, Jill Manthorpe, Charlotte Hanlon

**Affiliations:** 1 Department of Psychiatry, School of Medicine, College of Health Sciences, Addis Ababa University, Addis Ababa, Ethiopia; 2 Department of Inflammation Biology, Faculty of Life Sciences & Medicine, King’s College London, Weston Education Centre, 10, Cutcombe Road, London, SE5 9RJ UK; 3 Health &Social Care Workforce Research Unit, King’s College London, Strand, London, WC2 4LL UK; 4 Centre for Global Mental Health, Institute of Psychiatry, Psychology and Neurosciencem, King’s College London, 16 De Crespigny Park, London, SE5 8AF UK

**Keywords:** Realist review, Programme theory, Service user, Caregiver, Involvement, Mental health system

## Abstract

**Background:**

There is international recognition of the need for service user and caregiver involvement in mental health system strengthening. However, little is known about how best to integrate this approach into the mental healthcare system; what works to advance involvement, under what conditions, how and when does involvement bring added value, and how can it work in resource-poor settings in low and middle-income countries.

**Objective:**

To describe the methodology for a realist systematic review protocol to synthesise the evidence to explain the contexts, outcomes, and underlying mechanisms for involvement of service users with severe mental health problems and their caregivers in mental healthcare policy-making and planning, advocacy, service development, monitoring and improvement.

**Methods/designs:**

The proposed realist systematic review will involve five steps: (i) clarifying the review scope, (ii) a systematic search for evidence, (iii) evidence appraisal and data extraction, (iv) data analysis, (v) synthesis of evidence and formation of revised programme theory. Inputs from a formative qualitative study, consultative Theory of Change meetings with key stakeholder groups, and scoping reviews will be used to identify candidate theory/theories that will guide the selection, appraisal and analysis of studies, and refine the Theory of Change model that will be piloted and evaluated. Synthesis of data will be undertaken using realist logic, constant comparison and thematic analysis. In a consultative meeting with stakeholders the Theory of Change model will then be situated with respect to relevant programme theories and adapted to incorporate the synthesized evidence of relevance to the local context. The finalized Theory of Change model will be piloted and evaluated in a primary health care setting in rural Ethiopia.

**Discussion:**

Realist review methodology has not been applied to the area of mental health service user involvement in low- and middle-income country settings. In this protocol, we describe how this contextualized approach will be applied to identify and refine a theory-driven and transferable model of involvement of service users, embedded in ongoing work in Ethiopia.

*Systematic review registration* PROSPERO CRD42018084595

**Electronic supplementary material:**

The online version of this article (10.1186/s13033-018-0220-4) contains supplementary material, which is available to authorized users.

## Background

Service user and caregiver (SU/CG) involvement in mental health systems has become a mainstream policy expectation in many countries and has attracted growing research interest internationally [1–4]. SU/CG involvement in the mental health system pertains to policy making, strategic planning, service development and delivery, monitoring and evaluation or quality assurance, research, training and education, peer support and case management, and advocacy within the health system [4]. The involvement of SU/CGs can take place at the direct care or ‘micro’ level (e.g. individual care planning, assessment and case management), the health facility/community or ‘meso’ level (e.g. local service planning, service monitoring and evaluation), and the strategic or ‘macro’ level (e.g. policy making, strategic planning) [4–6], with the potential for different degrees of involvement.

There is wide recognition of the benefits of involving SU/CGs at all levels within mental health systems [7–9]. Involvement serves as a key indicator of democratization of health services, public accountability and transparency that can lead to more accessible and acceptable mental health services, enhance relevant service development, advance the culture and responsiveness of mental health services, and increase quality of care and cost-effectiveness [4, 7, 10–13]. SU/CG involvement can also improve health professionals` attitudes towards SU/CG and the relationships between service providers and SU/CG, as well as enhance SU treatment engagement, self-esteem and confidence, increasing satisfaction with mental health care, and helping to empower SUs to gain control over their own recovery [4, 7, 11, 14]. In low and middle-income countries (LMICs), SU/CG involvement has been proposed as an essential means of strengthening weak mental health care systems [3, 15] and promote equitable scale up of respectful and quality mental health care [16, 17].

Despite the rhetoric and the wide recognition of the value of SU/CG involvement in strengthening mental health systems in policy documents and academic literature, involvement is limited. A gap remains about how meaningfully to involve SU/CG, the impact of their involvement in different contexts, when and how participation works, and why [4, 7, 18]. In LMICs, SU/CG contributions to the mental health system have received minimal attention and the health systems often fail to meet the needs of people with mental health problems [19–21]. SUs are commonly excluded from their rights towards full citizenship and from meaningful participation in decisions that directly affect them [18, 20, 21].

However, SU/CG involvement is a complex process, a multifaceted construct with multiple meanings, involving different approaches, various levels, and numerous processes [4, 6, 22, 23]. There is a lack of consensus about what precisely SU/CG involvement means; different terms (e.g. patient/caregiver engagement/co-production, consumer/family participation, patient and public involvement) are used and defined in the literature [4, 8, 22]. For the purpose of this study we adapted from Tambuyzer et al. [4] and Carman et al. [5] the following definition for the term or concept “service user/caregiver involvement”:

‘‘*… the active and meaningful involvement by service user, caregivers, and their representatives in decision*-*making and participation in a range of activities (e.g. policy making, planning, service development and delivery, quality improvement, monitoring and evaluation, research, education and training) starting from the ‘expertise by experience’ of the person, in collaboration with and as equal partners of professionals to improve health and health care quality’’*.

Systematic reviews of SU/CG involvement in mental health have usefully described the context, type, barriers and facilitators to involvement, general lessons for good practice, and to a lesser extent the impact of involvement [4, 7, 11, 12, 18]. However, these reviews focused on the methodological qualities of studies and were not designed to disentangle or offer theoretical explanations about the complex causal relationships that exist between SU/CG involvement components, contexts and outcomes of SU/CG involvement, for whom, and why [24, 25]. As realist review methodology has not been applied to the area of mental health service user involvement in low- and middle-income countries, in this protocol paper we describe how this contextualized approach will be applied to identify and refine a theory-driven and transferable model of involvement of service users and caregivers.

## Objective

The general objective of the proposed study is to synthesize evidence about the theoretical and empirical basis of involvement strategies for SUs with severe mental disorders and their CGs to inform the development, testing and evaluation of Theory of Change model (ToC) underpinned by programme theory for integration of SU/CG involvement in mental healthcare scale-up within primary health care setting in rural Ethiopia.

The specific objectives of the proposed realist review are to:

- develop a range of initial programme theories, and draft a ToC model that describe how SU/CG involvement works, for whom, in what circumstances and why;
- synthesise evidence on the contextual factors, mechanisms and outcomes of existing models and strategies for involvement of SU with severe mental disorders and their CG;
- refine the programme theory or theories that explain the relationships between the mechanisms underlying these involvement strategies, the particular outcomes of the mechanisms and the contextual factors that may influence these relations; and
- use the programme theory and evidence synthesis to inform the locally embedded ToC model, and test and evaluate the resulting model for the integration of SU/CG involvement in a rural LMICs setting.

## Methods

### Design: realist review

Given the complexity of SU/CG involvement, a realist review can offer key methodological tools and the theoretical basis to facilitate the exploration of learning, accumulation of evidence and the transfer of lessons in different contexts [26–28]. The realist review approach is grounded in realism (a realist philosophy of science) [29–32]. The principles of realism states that (i) causal explanations are achievable; (ii) social reality is mainly an interpretive reality of social agents/actors; and (iii) social agents/actors evaluate their social reality [30, 31, 33, 34]. In a realist review, the aim is to explain how complex programmes operate or not, the underlying theory (theories) of intervention (in this case strategies and models for involvement), the interaction with the intervention context (C), the causal mechanisms (M), and how mechanisms produce different patterns of outcomes (O) in different contexts [24, 28, 29, 35, 36]. In this way, a realist review helps to synthesize a broad range of theoretical insights and qualitative, quantitative and mixed methods research evidence in the form of a programme theory for complex interventions applied in different settings [28, 29, 37]. As the context–mechanism–outcome (CMO) configuration emerges, insights accumulate and can attain the level of a so-called middle-range theory (MRT); and the process can build on programme logic models (for example, ToC) that define the components, mechanisms of action and outcomes of specific interventions [37, 38]. Accordingly, the focus of a realist review is on building, testing and refining programme theory or theories regarding complex causal mechanisms and how these interact with individuals’ agency and social context to produce outcomes [36].

**Fig. 1:**
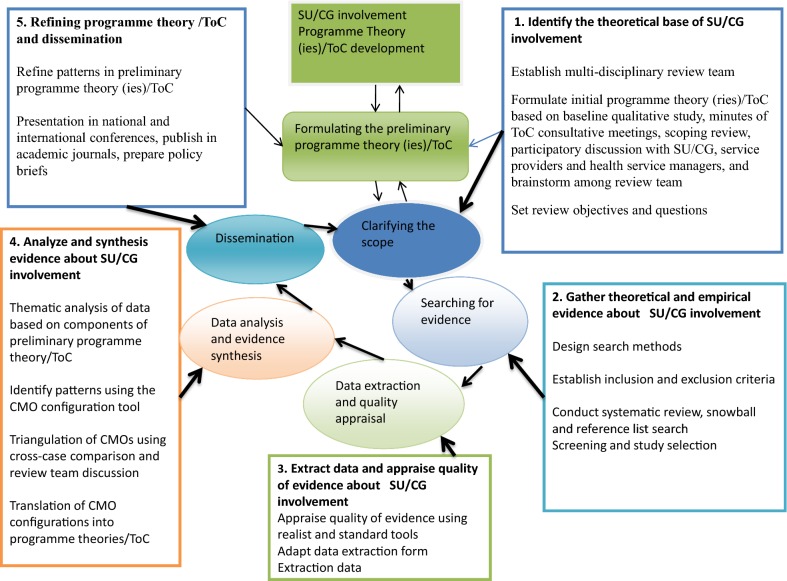
Flow chart for realist review and synthesis of evidence (adapted from Pawson and Tilley [30], and Marchal et al. [42])

For this proposed study, realist syntheses can offer methodological tools to build on the evidence from conventional reviews, other observational studies, input from stakeholders, and the experience of the review team to capture the complexity of SU/CG involvement and explore the “missing links” [39] or limitations of conventional systematic reviews [7, 11, 18, 40]; specifically, “*what works best, how, for whom and when and why, under what conditions*” [24, 36, 41]. A realist synthesis can help to identify a range of MRTs that guide programme theory and a ToC map that can be populated with evidence, refined, tested and evaluated in the study’s local context [24, 29, 36, 37, 41]. The refined theory and ToC can provide rich contextual information and explanation of how and why integration of SU/CG involvement improves a mental healthcare system. The model can be applied by healthcare stakeholders to guide real-world decision making with transferable understanding of mechanisms [24]. Hence, the authors will employ a five-step realist review method to gain contextualized understanding of how and why integration of SU/CG involvement is practiced and what mechanisms lead to mental health system strengthening in realist concepts and terminology [24, 27] (see Additional file 1, for definitions of concepts and terms). In the next section the five steps are presented with detailed descriptions, and are also illustrated in Fig. 1.

### Step 1: clarifying scope

In step 1, the initial programme theory (theories) review questions and search terms will be identified based on a ToC map developed through analysis of experiential knowledge of stakeholders that sets out a framework and explanation about SU/CG interventions, outcomes, assumptions and indicators. This step will help to conduct focused reviews of the literature to examine and synthesize evidence [27, 41]. This review stage will be achieved through a mixture of iterative methods presented below.

A review team will be established, with participants from a variety of disciplinary backgrounds (public health, psychology, medical sociology, social work, psychiatry, and mental health research). The role of the review team will be to draw on varied conceptualizations and experience of SU/CG involvement and to integrate distinct methodological approaches into a programme theory that takes into account the complexity of SU/CG involvement.

Realists suggest tapping into stakeholders and experts as an initial strategy to help clarify the scope of the question and identify theories that may explain successful SU/CG involvement in LMICs [24]. In line with this, the review team will use the two information sources from a rural Ethiopian context to explore candidate theories and develop a draft ToC; (i) a formative qualitative study conducted on the experience, barriers, facilitators and capacity building needs with key stakeholders (policy makers/planners, health center heads, SUs and CGs) [20], and (ii) the minutes/notes of consultative meetings with senior psychiatrists and researchers, and key stakeholders in Sodo district, rural Ethiopia, about how to integrate SU/CG involvement in mental health system strengthening. The baseline qualitative study identified multi-level barriers (strategic, healthcare, community and service user) and potential strategies to overcome them. This helped to enrich the Theory of Change (ToC) road map developed with stakeholder groups. The draft ToC map comprises interventions for: (i) local community members, (ii) health care providers and managers, (iii) service users, and (iv) caregivers. For each intervention, distinct preconditions (intermediate outcomes), assumptions and indicators were specified. The components of the ToC map will provide the starting points for discussion and consensus regarding the initial theoretical basis for the scoping review, development of “initial theories” [41], focus the review questions and guide the selection of search terms and strategies. At this stage, a consultative session within the review team will be facilitated to identify candidate theories related to the research objectives. The review team members will be asked to provide the most relevant literature that describe theoretical underpinnings and share their expertise about what, how and why involvement strategies work, generally and within the context of SU/CG involvement in mental health systems. Moreover, a scoping search will be undertaken of the peer-reviewed and grey literature [27]. Finally, based on the above gathered data the lead reviewer will establish a working list of candidate programme theories, circulate the candidate theories to the review team to identify and select amongst the potential theories, and provide a “reality check” on the clarity and explanatory strength of the selected theories. Through brainstorming with the review team to create the initial CMO configurations, a consensus on the candidate theories and how these can inform the review questions will be established. Generic questions will be created, and the initial programme theories (which will undergo a number of iteration and refinement) will be applied to guide the review, data extraction, analysis and synthesis processes to understand the context–mechanism–outcome (C–M–O) relationship for integration of SU/CG involvement in mental health systems [41].

In addition, a participatory action-oriented training workshop will be conducted with SUs, CGs, service providers, and health facility managers in Sodo district. The initial programme theories will be discussed with SU, CG and service providers/mangers to get their inputs and participation. The participants will be invited to provide inputs about (i) how to integrate SU/CG involvement in a mental health system, (ii) what type and level of involvement SU/CGs would like to achieve and, (iii) draft strategies and action plans to operationalise SU/CG involvement for the local contexts. These inputs will guide relevant theories and establish a preliminary contextualized draft of ToC involvement.

### Step 2: searching for evidence

Following the development of candidate programme theories and a draft ToC, an extensive purposive search of peer-reviewed literature will be undertaken by two review team members to seek suitable evidence to refine these candidate theories and ToC. The search methodology will be informed by the standards and guidelines for realist syntheses [41]. Details of search methods, terms, inclusion and exclusion criteria, screening and selection of studies are presented below.

#### Search methods

The search method will be purposive, focused on articles on SU/CG involvement in mental health systems in both high income and LMICs. A recent systematic review focusing on LMICs [18] found that publications on SU/CG involvement were limited; therefore, the scope of this review will be expanded to include other countries. The first search will be conducted by two reviewers of PubMed, EMBASE, PsycINFO, and CINAHL databases for the period 2002 to the search date. This will be supplemented by any earlier papers identified through lateral searches. As the search is purposive, search terms will be produced for each of the candidate theories and components of the draft ToC. Some initial search terms were developed by the lead reviewer following previous systematic reviews [4, 18]. The final list of terms to guide the search will be determined in discussion with other team members. To capture relevant articles, the search will be carried out using various combinations of MeSH terms and free text with variants of the following four domains: (i) SU/CG involvement (including terms such as patient and carer), (ii) mental disorders, (iii) mental health systems, and (iv) models/framework. The four domains will be combined using Boolean operator ‘AND’ and applied to the above databases (see Additional file 2 for details of initial search terms that will be adapted for each database).

To capture grey literature and additional citations/articles, hand searching of reference lists/reference scanning will be performed, including citation links within the literature and on Google and Google Scholar. In addition, discussion will be conducted within the review team to optimize the knowledge and networks of the research team about relevant publications in their specific areas of specialization; and also consult experts. After the initial search, a secondary search will be performed based on the depth and comprehensiveness of literature collected. All identified sources will be uploaded into Endnote X7 [43] for citation management.

#### Inclusion and exclusion criteria

In keeping with the nature of the realist review [24, 29, 36, 37], all publications (qualitative, quantitative or mixed method) that report adult SU/CG involvement in mental health systems will be considered. Both empirical and theoretical documents from peer reviewed and grey literature that provides information about the context, mechanisms and outcomes of the involvement will be included. The specific inclusion and exclusion criteria that guide the screening and selection of documents are presented in Table 1.

**Table 1:** Inclusion and exclusion criteria

Inclusion criteria	Exclusion criteria
Service user/caregiver involvement within mental health care as main focus Includes psychiatric conditions with an emphasis on severe mental disorders (including psychosis, schizophrenia, major depression, bipolar disorder) and their caregivers Service user/caregiver involvement in mental health systems Written in English language Adults of age 18 years and above LMICs and high income countries (no geographical limitations)	SU/CG involvement outside of health care covering non-psychiatric conditions and mental disorders other than severe mental disorders Service user/caregiver involvement in their own treatment and care, without broader system level involvement User/caregiver involvement as respondents of research, information and consultations/opinion giving Written in languages other than English Participants below age of 18 years

#### Screening and selection of studies

Screening and selection of articles will take place in two stages (title and abstract, and full text) [41]. The lead reviewer will read the titles and abstracts, and apply the inclusion and exclusion criteria to decide if the full articles should be retrieved and then read the full texts. To reduce bias, two other reviewers will independently screen 10% of randomly selected studies [44] and cross-check results, establish consensus on the relevance of the documents and resolve any disagreement. The whole process of study selection will be guided by the use of the preferred reporting items for systematic reviews and meta-analyses (PRISMA) flow diagram [45].

### Quality appraisal and data extraction

Following the screening, the quality of the selected articles will be assessed to determine the credibility of findings and theoretical assertions found in each study. Data will then be extracted.

#### Quality appraisal

Realist reviews usually draw on evidence from a wider range of sources than traditional systematic reviews (that value procedural uniformity and methodological quality mostly focusing on primary studies), and employ various techniques for assessment of quality of evidence [29, 36]. In this particular study, quality of evidence will be assessed in two ways. First, quality appraisal in a realist synthesis is not limited to the hierarchy of evidence or the methodological quality of the study; rather, each document/study is assessed based on its applicability to the theory in question, and methodological appropriateness in relation to the credibility and trustworthiness of the approach [29, 41]. Consistent with a realist synthesis approach, documents/papers will be assessed iteratively within the review team to determine whether the evidence provided is considered “*good enough and relevant enough*” (see Additional file 1 for description) [29, 46, 47] to inform the understandings of SU/CG involvement strategies and their respective CMO configurations [29, 37, 41]. After checking relevance, a hybrid classification tool will be applied to categorise studies that are conceptually thick (rich), or thin (weaker) [48, 49] cited in [50] (see Additional file 1 for description). A hybrid appraisal tool has been found to be practical and useful in theory-driven reviews as it enables reviewers to focus on the stronger sources of programme theories without excluding weaker sources that may make an important contribution [51].

Second, the quality of the studies will be assessed using standard quality assessment tools to “*illuminate the richest picture*” [27] to ensure transparency, validity, reliability and verifiability of findings and conclusions [52]. Accordingly, for qualitative studies and non-randomized studies, the Wallace criteria [53] will be employed, and for randomized studies, the Cochrane Collaboration’s tool for assessing risk of bias [54] will be applied. Careful consideration will be taken not to exclude studies/documents based on methodological rigor alone, because they may usefully have explored a very specific hypotheses about the relationships between context, mechanism, and outcomes [27].

#### Data extraction

Data extraction will be conducted on the basis of relevance to the agreed review questions and will be based on realist guidelines that differ from typical population, intervention, comparison, outcome (PICO) questions and instead ask “what is it about SU/CG involvement that works, for whom, in what circumstances, in what aspects (or where), and why?” [37, 55]. A matrix will be created, similar to that used in a previous realist synthesis [37]. Accordingly, the lead reviewer will develop the data extraction form to gather information about CMO configuration, contextual information, study characteristics (e.g., authors, publication data, study design, empirical or theoretical, geography), SU/CG characteristics (e.g. age, gender, diagnostic category), characteristics of interventions/strategies (e.g. types of interventions, mental health system component, level of involvement), implementation context (e.g. barriers, facilitators, settings) and outcomes (e.g. at SU/CG level, health facility, health system).

The extraction records will be managed using a Microsoft Excel spreadsheet. The lead reviewer and two other reviewers will pilot this extraction form, read in detail and extract information independently on a sample of publications, and the results will be discussed between the team to refine the extraction form. A codebook will assist to ensure shared understanding of concepts. Based on the content of the codebook and data extraction form the lead reviewer will read all selected articles, and extract descriptive study characteristics. A 10% random subsample of coded articles/documents will be reviewed by three other reviewers for consistency. Disagreement will be resolved through discussion [44].

### Analysis and synthesis

At this stage, various iterative methods will be employed to analyze and synthesis the data extracted and use a realist logic to interrogate the programme theory/ToC: first, regroup the data extracted by the reviewers independently from the included studies/documents to build amalgamated case summaries in a single table that will provide a rich description of SU/CG involvement in the mental health system.

Second, through the application of a mix of deductive, inductive, retroductive and abductive analytical processes, each paper will be examined for evidence based on how the evidence supports, refutes, reinterprets or refocuses our initial programme theory/ToC via a thematic analysis approach [56], as a first stage analysis. Deductively, the initial programme theory/ToC model components will guide the emergence of the themes. Three reviewers will independently analyze the studies/documents; code passages of articles/documents related to the initial programme theories and ToC components. It is anticipated that the data extraction process, using the initial programme theories and components of the ToC, may also inductively lead to identification of emerging themes. The employment of abductive and retroductive inferences will help to annotate passages of text which disconfirm our initial programme theory/components of ToC or which mention important elements of involvement that fall outside these theory components [57]. To guarantee consistency, trustworthiness and connection between the extracted data and the themes will be examined, discussed and repeatedly tested during the coding and analysis process by the review team.

Third, during the coding and data organizing process, the coded extract that refers to the specific context, mechanism or outcome will be determined, and how the configuration of CMO contributes to our programme theory/ToC [41]. The main themes will be arranged according to their reference to context, interventions (in this case involvement), outcomes and mechanisms to develop CMO configurations [29]. For each paper/document, the specific CMO configuration and a draft narrative synthesis will be developed. Using abductive and retroductive inferences, new relationships between the context, mechanisms and outcomes associated with each SU/CG involvement will be considered. We anticipate that this will allow for multiple CMOs.

Finally, a pattern for CMO configurations will be identified using a mix of abduction, retroduction [57], constant comparative analysis [58] and realist review logics [27, 29]. The data will then be synthesised through a process of reasoning that is structured around: juxtaposition, reconciling, adjudication, consolidation, and situating [27, 29, 41] (see Additional file 1, for descriptions of concepts and terms). Accordingly, patterns in CMO (demi-regularities or semi-predictable patterns) and their commonalties will be identified, summarized and tested by confronting them with the data of each case to check their explanatory power. The initial programme theories/ToC will be refined and finalized for piloting. The refined programme theories/ToC will be discussed and finalized with the entire review team. The review will be reported in accordance to the Realist And MEtanarrative Evidence Syntheses: Evolving Standards (RAMESES) publication standards [37].

## Dissemination

Once the revised programme theory/ToC has been developed, a presentation will be arranged with health professionals, health managers and people who are, or who have been, service users and caregivers, to gather their insights about the ToC model. The results of the realist review will be submitted to peer-reviewed academic journals and also presented during national and international conferences, including a briefing document for health system managers and SU/CG organisations.

## Discussion

SU/CG involvement has generated interest as a means to strengthen mental health systems globally. Involvement is associated with various potential benefits for SUs, CGs, service providers, health facilities and the health system in general. However, it has been argued that not nearly enough is being put in place to sustainably involve SUs/CGs in the healthcare system [4, 18, 59]. Involvement of these partners is not yet recognized as a key component of healthcare systems, particularly in LMICs [18]. Integration of SU/CG involvement in a mental health system is a complex process, influenced by the interplay of SU/CG, service provider, health facility and health system factors [4, 6, 20]. This may be the reason why practical implementation and development of strategies/models on how best to involve SU/CGs have been limited [4, 12, 18]. In addition, many of the challenges to integrating SU/CG involvement can be also explained by the lack of value attributed to the advantage of informal (knowledge from experience) and formal theories in planning and executing the involvement efforts [60, 61].

### Strengths and challenges

Realist review can provide a sound theory–driven evidence for model development and identify where gaps in the evidence may lie.

However, some researchers have raised questions about the nature of the mechanisms and the challenges of differentiating between mechanisms and essential context conditions [62]. To overcome this challenge discussion will be held to reach some consensus on key terms and concepts between reviewers, and codebooks will be developed during data extraction and coding processes. Compared to conventional systematic reviews, in realist reviews it is a challenge to reproduce the review because of the utilisation of a mixture of evidence and processes [36, 55]. We will reduce the impact of this challenge and will develop a summary table as well as present (i) methodological details and (ii) our findings to clearly illustrate how through each step the team will have arrived at our conclusions.

## Conclusions

In this project the utilization of the combination of informal knowledge (lived experience), formal theories [60] and reviews of peer-reviewed and grey literature may be a step in the right direction. Synthesizes of evidence through a realist review is complex, underutilized and relatively neglected in the area of SU/CG involvement, but may have potential for strengthening mental health systems, particularly in LMICs. This relatively new approach to evidence synthesis that incorporates contexts, mechanisms and outcomes of SU/CG involvement through current literature and key stakeholders’ input and that of the review team, will expand current knowledge on how best to integrate involvement of SU/CG within mental health systems. The next step of the proposed study will be to conduct the review, refine, test and evaluate ToC for integration of SU/CG within primary health care setting in rural Ethiopia.

## Additional files

**Additional file 1.** Definition of realist review key concepts and terms used in this study, adapted from [1–6].

**Additional file 2.** Initial search terms to inform search strategy (Service user and caregiver domain) AND (mental disorders domain) AND (health system domains) AND (Models domains).

## Abbreviations

CMO: context–mechanism–outcome
LMICs: low- and middle-income countries
Emerald: emerging mental health systems in low- and middle-income countries
MRT: middle-range theory
SU/CG: service user and caregivers
ToC: Theory of Change model
