# Supplementary material for: Development of programme theory for integration of service user and caregiver involvement in mental health system strengthening: protocol for realist systematic review
Source: Int J Ment Health Syst. 2018 Jul 24;12:41. doi: 10.1186/s13033-018-0220-4 (PMC6057009; doi:10.1186/s13033-018-0220-4)
Supplement: Supplementary file 1 — Additional file 1. Definition of realist review key concepts and terms used in this study, adapted from [1–6]. [file 13033_2018_220_MOESM1_ESM.docx]

**Additional file 1**: Definition of realist review key concepts and terms used in this study, adapted from [[1-6](#_ENREF_1)]

| Key concepts | Description |
| --- | --- |
| Context | The wider configuration of factors, not necessarily connected to a programme, which may enable or constrain the operation of specific mechanisms  Also refers to the circumstances in which the programme is implemented including features of the health facilities and health care system, staffing, culture that are necessary for the org mechanism to lead to outcomes |
| Mechanisms | Refers to a generative force triggered in particular contexts that lead to outcomes. It often denotes the reason (cognitive or emotional) of the various actors, i.e. people with mental illness, their caregivers, service providers, local community and those within health system  Mechanisms are linked to, but are not the same a, a service`s strategies or interventions. Identifying the mechanisms goes beyond describing “ what happened” to theorizing “why it happened, for whom and under what circumstances”  How a programme resource or opportunities interact with the reasoning of individuals( beliefs, values, desires, cognitive process) and lead to changes in behavior or produce outcomes |
| Outcome | The outcome is a result of the interaction between a mechanism and its triggering context. Outcomes may include service user and caregivers greater engagement in mental health system components  Include short-term, medium-term and long-term changes(on service user, caregivers, service providers, health facility and health care system) intended and unintended ,resulting from the intervention(in this case service user and caregiver involvement) |
| CMO configuration | Conceptual tool to link the elements of context, mechanisms and outcomes of an intervention |
| Programme theory | Those ideas about what needs to be changed or improved in how service users and caregivers are integrated in mental health system, what needs to be in place to achieve improvements(s) and how programmes are believed to work. The programme theory specifies what is being investigated and the elements and scope of the review  Set of hypotheses that explain how and why service user and caregiver involvement, strategies/ models of involvement are expected to produce outcomes. These contain, even if they do not explicitly state, ideas about how a problem can be best addressed and how factors that may undermine the actions of a programme can be themselves be addressed.  Also refers to an abstracted description and or diagram that lays out what a program or family of programmes or interventions comprise and how it is expected to work. |
| Middle-range theory | Formal published theories that provide the initial rationale for design and implementation of the intervention. Level of theoretical abstraction that provides an explanation of semi-regularities in the CMO interactions or a set of interventions. |
| Demi-regularity | semi-predictable pattern or pathway of programme functioning |
| Juxtaposition of sources of evidence | To place two or more things(evidence fragments) together, especially in order to suggest a link between them or emphasis the contrast between them, for example, when evidence about implementation in one source enables insights into evidence about outcomes in another source |
| Reconciling of source of evidence | To make two or more apparently conflicting things (evidence fragments) consistent or comparable. Where results differ in apparently similar circumstances, further investigation is appropriate in order to find explanations for why these different results occurred |
| Adjudication of sources of evidence | To make a judgment about methodological quality or applicability in this instance and account for this judgment based on findings from the use standard quality appraisal tools or explicit argument about why a piece of evidence was not applicable. In short, making comparison on the basis of methodological strengths or weaknesses |
| Consolidation of sources of evidence | To bring together, when evidence about mechanisms and outcomes is complementary and enables a multi-faceted explanation to be built into a more coherent whole |
| Situated sources of evidence | To place something (a piece of evidence) in a context or set of circumstances and show the connections (between it/ them and other evidence fragments). This is, where outcomes differ in particular contexts, an explanation can be constructed of how and why these outcomes occur differently |
| Quality assessment | ‘Good enough’: is based on the quality of evidence, for example whether it is of a sufficient standard for the type of research, and whether the claims made are considered to be trustworthy [[2](#_ENREF_2), [6](#_ENREF_6), [7](#_ENREF_7)]  ‘Relevant enough’: related to whether the authors provided sufficient descriptive detail and/or theoretical discussion to contribute to the theories generate [[2](#_ENREF_2), [6](#_ENREF_6), [7](#_ENREF_7)]  **Conceptually rich**: theoretical concepts are unambiguous and described in sufficient depth to be useful  Relationships between and among concepts are clearly articulated  Concepts sufficiently developed and defined to enable understanding without the reader needing to have first-hand experience of an area of practice  Concepts grounded strongly in a cited body of literature  Concepts are parsimonious( i.e. provide the simplest, but not over-simplified explanation[[8](#_ENREF_8)] in [[9](#_ENREF_9)] p.28  **Thicker description but not conceptually rich**: description of the programme theory or sufficient information to enable it to be ‘surfaced’  Consideration of the context in which the programme took place  Discussion of the differences between programme theory( the design and operation of programme-what was intended) and implementation( what ‘happened in real life’)  Recognition and discussion of the strengths and weaknesses of the programme as implemented  Some attempt to explaining anomalous results and findings with reference to context and data  Description of the factors affecting implementation[[10](#_ENREF_10)] in [[9](#_ENREF_9)] p.28  **Thinner description**: insufficient information to enable the programme theory to be ‘surfaced’  Limited or no consideration of the context in which the programme took place  Limited or no discussion of the difference between programme theory( the design and orientation of a programme-what was intended) and implementation( what ‘happened in real life’)  Limited or no discussion of the strengths and weaknesses of the programme as implemented  No attempt to explain anomalous results and findings with reference to context and data  Limited or no description of the factors affecting implementation[[10](#_ENREF_10)] in [[9](#_ENREF_9)] p.28 |
|  |  |

References

1. Pearson M, Chilton R, Woods HB, Wyatt K, Ford T, Abraham C, Anderson R: **Implementing health promotion in schools: protocol for a realist systematic review of research and experience in the United Kingdom (UK)**. *Systematic reviews* 2012, **1**(1):48.

2. Pawson R: **Evidence-based Policy: A Realist Perspective**. London: Sage; 2006.

3. Davidoff F, Dixon-Woods M, Leviton L, Michie S: **Demystifying theory and its use in improvement**. *BMJ Qual Saf* 2015:bmjqs-2014-003627.

4. Wong G, Greenhalgh T, Westhorp G, Buckingham J, Pawson R: **RAMESES publication standards: realist syntheses**. *BMC Medicine* 2013, **11**(1):21.

5. Goicolea I, Hurtig A-K, San Sebastian M, Vives-Cases C, Marchal B: **Developing a programme theory to explain how primary health care teams learn to respond to intimate partner violence: a realist case-study**. *BMC Health Services Research* 2015, **15**(1):228.

6. Bunn F, Goodman C, Malone JR, Jones PR, Burton C, Rait G, Trivedi D, Bayer A, Sinclair A: **Managing diabetes in people with dementia: protocol for a realist review**. *Systematic Reviews* 2016, **5**(1):5.

7. Rycroft-Malone J, Burton C, Hall B, McCormack B, Nutley S, Seddon D, Williams L: **Improving skills and care standards in the support workforce for older people: a realist review**. *BMJ Open* 2014, **4**(5):e005356.

8. Ritzer G: **Metatheorizing in sociology. Lexinton, MA: Lexington Books;**. 1991:113-131.

9. Pearson M, Hunt H, Cooper C, Shepperd S, Pawson R, Anderson R: **Intermediate care: a realist review and conceptual framework**. *Final Report Southampton: NIHR Service Delivery and Organisation programme* 2013.

10. Roen K, Arai L, Roberts H, Popay J: **Extending systematic reviews to include evidence on implementation: methodological work on a review of community-based initiatives to prevent injuries**. *Social Science & Medicine* 2006, **63**(4):1060-1071.
