# Supplementary material for: Development of programme theory for integration of service user and caregiver involvement in mental health system strengthening: protocol for realist systematic review
Source: Int J Ment Health Syst. 2018 Jul 24;12:41. doi: 10.1186/s13033-018-0220-4 (PMC6057009; doi:10.1186/s13033-018-0220-4)
Supplement: Supplementary file 2 — Additional file 2. Initial search terms to inform search strategy (Service user and caregiver domain) AND (mental disorders domain) AND (health system domains) AND (Models domains). [file 13033_2018_220_MOESM2_ESM.docx]

Additional file 2: Initial search terms to inform search strategy (Service user and caregiver domain) AND (mental disorders domain) AND (health system domains) AND (Models domains)

| Service user and caregiver involvement domain terms( all will be combined with OR) | Mental disorders domain terms( all will be combined with OR) | Health system domain terms ( all will  be combined with OR) | Models domains( all will  be combined with OR) |
| --- | --- | --- | --- |
| “patient participation”  “patient outcome assessment”  “patient-centered care”  “community participation”  “patient engagement”  “patient involvement”  “patient empowerment”  “patient collaboration”  “ex-patient engagement”  “public and patient involvement”  “service user engagement”  “service user involvement”  “service user participation”  “user engagement”  “user participation”  “user empowerment”  “consumer participation”  “consumer involvement”  “consumer engagement”  “consumer empowerment”  “client participation”  “citizen involvement”  “citizen engagement”  “expert by experience involvement” “survivor involvement”  “recipients involvement”  “people affected with mental illness involvement”  “ lay person engagement”  “family engagement”  “family involvement”  “family participation”  “caregiver participation”  “caregiver involvement”  “caregiver engagement”  “carer involvement”  “carer participation”  “carer engagement  Patient-centerd  Shared-deciosion* | “Mental health”  “mental problems”  “mental disease”  “severe mental illness”  “serious mental illness”  “psychiatric illness”  Schizophrenic  schizoaffective  “mental disorders” “mentally ill persons”  Psychoses  “major depressive disorder”  schizophrenia  “ psychotic disorders” “bipolar disorder”  “mood disorder”  “affective disorders”  “people with a psychiatric diagnosis”  “people with mental disorders”  “people with mental health conditions”  “psychiatric survivors” | “Delivery of health care” “Health policy”  “policy making”  “health care reform”  “Health services”  “Mental health services”  “community mental health services”  “community health planning”  “health planning”  “health services research”  “public health systems”  research  “outcome assessment”  “quality control”  “quality assurance”  “quality of health care”  “self-help groups”  “peer group”  “consumer organizations”  “patient care planning”  “advance care planning”  “decision making”  “self-care”  “case management”  “patient care planning”  “mental health associations”  “patient advocacy”  “consumer advocacy”  “Community mental health  planning  “mental healthcare delivery”  “Health system strengthening” “Mental health policy”  “Mental health system” “mental health research” “mental health strategies”  “development of services” “training of health workers”  “education of health workers”  “monitoring and evaluation”  “mental health self-help”  “peer support”  “consumer run-organizations”  “mental healthcare planning”  “shared-decision making” | Models  Frameworks  Strategies  Guidelines  Roadmaps  Interventions  Theory  Theories  Capacity Building  Intervention  Training |
